# FACTORS ASSOCIATED WITH LIFE-SPACE CONSTRICTION IN LATER LIFE: EVIDENCE FROM THE HEALTH AND RETIREMENT STUDY

**DOI:** 10.1093/geroni/igz038.1917

**Published:** 2019-11-08

**Authors:** Moon Choi, Briana Mezuk

**Affiliations:** 1 KAIST Graduate School of Science and Technology Policy, Daejeon, Korea, Republic of; 2 University of Michigan School of Public Health, Ann Arbor, Michigan, United States

## Abstract

This study aimed to examine factors associated with life-space constriction, using the data from the Health and Retirement Study, a nationally representative sample. We limited our analysis to those who were 65 years and older and answered to the 2012 experimental module on life-space (N=895; mean age=75.3; 59.4% women). Life-space was assessed with the modified version of the UAB Study of Aging Life-Space Assessment, ranging nine zones: room, home, own property, immediate neighborhood, town, community, county, state, and region. A series of logistic regression models were used to estimate odds ratios for life-space constriction by sociodemographic and health characteristics. The results showed that 3.0% and 6.7% of older adults reported that they had never been to places beyond their home and own property/apartment building for the past four weeks, i.e. the critical boundaries in terms of social isolation. The significant factor associated with the life-space constriction within home, immediate neighborhood, and town was physical mobility limitation (OR: 1.18, 1.09, 1.11, respectively), while the constriction within county was associated with education level (OR: 0.91). Driving a car was negatively associated with the life-space constriction within own property/apartment building and home (OR: 0.48 and 0.22, respectively). Policy makers need to pay more attention to social and environmental factors influencing social isolation among older adults such as transportation options and social class disparity.

